# Decoding Hyponatremia: A Systematic Review of Diagnostic Pathways and Therapeutic Approaches Applied When Correction Fails

**DOI:** 10.7759/cureus.96131

**Published:** 2025-11-05

**Authors:** Kiranjot Kaur, Sharmila Venkatachalapathi, Mashal Mumtaz, Muhammad Faizan Butt, Mirna Morad Mashhour, Shashwat Shetty, Maryam Zafar, Mohammed Usman Khan Wazir, Neiloofar Mohmand, Ayesha Farooqi

**Affiliations:** 1 United States Navy, United States Armed Forces, Great Lakes, USA; 2 Clinical Research, Arizona State University, Tempa, USA; 3 Medicine, Shri B. M. Patil Medical College, Bijapur, IND; 4 Internal Medicine, Periyar Government District Head Quarters Hospital, Mayiladuthurai, IND; 5 Internal Medicine, Vadamalayan Hospitals Pvt Ltd., Dindigul, IND; 6 Internal Medicine, University College of Medicine and Dentistry, Lahore, PAK; 7 Gastroenterology, NHS Forth Valley, Larbert, GBR; 8 Haematology, Sheffield Teaching Hospitals NHS Foundation Trust, Sheffield, GBR; 9 Medical Education, NHS Highland, Inverness, GBR; 10 Internal Medicine, Banha Health Insurance Hospital, Banha, EGY; 11 Orthopaedics, Hillingdon Hospital, Uxbridge, GBR; 12 Paediatrics, Basildon University Hospital, Basildon, GBR; 13 Trauma and Orthopaedics Surgery, Northwick Park Hospital, Harrow, GBR; 14 Internal Medicine, Kabul University of Medical Sciences "Abu Ali Ibn Sina", Kabul, AFG; 15 General Surgery, Dr. Ruth K. M. Pfau Civil Hospital Karachi, Dow University of Health Sciences, Karachi, PAK

**Keywords:** diagnostic pathways, electrolyte disorders, hyponatremia, refractory hyponatremia, siadh, vasopressin receptor antagonists, water balance

## Abstract

Hyponatremia is the most common electrolyte disorder encountered in clinical practice. Conventional management strategies, including fluid restriction, hypertonic saline, and solute supplementation, often fail to achieve sustained correction, particularly in the syndrome of inappropriate antidiuretic hormone secretion (SIADH), resulting in refractory cases. A systematic search was conducted according to the Preferred Reporting Items for Systematic Reviews and Meta-Analyses (PRISMA) 2020 guidelines. Eligible studies included randomized controlled trials, cohort studies, guideline syntheses, and mechanistic research, with risk of bias carefully evaluated. Seven studies met inclusion criteria. Tolvaptan consistently improved serum sodium concentrations in patients with SIADH and heart failure, though its use was limited by high cost and the potential for overly rapid correction. Oral urea emerged as a safe, inexpensive, and reliable option for gradual, sustained normalization of sodium levels. Vasopressin receptor antagonists (vaptans) demonstrated superiority over fluid restriction in oncology-associated hyponatremia, although relapse was frequently observed following discontinuation. Demeclocycline exhibited mechanistic efficacy but remains limited by nephrotoxicity risk. Guideline-based analyses emphasized improved diagnostic precision but revealed inconsistencies in correction thresholds and recommendations for second-line therapy. When standard correction measures fail, clinicians should reassess the underlying etiology and escalate treatment accordingly. Vaptans offer rapid correction, urea ensures safe long-term management, and demeclocycline serves as a last-line therapeutic option.

## Introduction and background

Hyponatremia, defined as a serum sodium concentration below 135 mmol/L, is the most common electrolyte disorder in hospitalized patients. Its prevalence has been estimated at 5% in the community and up to 30-35% among inpatients [1]. Even mild or chronic hyponatremia is clinically significant, as it is associated with gait disturbances, cognitive impairment, fractures, and increased mortality risk [2]. These findings highlight the importance of not only recognizing hyponatremia but also implementing effective corrective strategies to improve patient outcomes. Conventional management is guided by underlying volume status. In hypovolemic hyponatremia, isotonic saline infusion restores intravascular volume and suppresses non-osmotic antidiuretic hormone (ADH) release [3].

Hypervolemic hyponatremia, commonly encountered in congestive heart failure or cirrhosis, is generally managed with fluid and sodium restriction together with loop diuretics and careful volume management [4,5]. In euvolemic hyponatremia, especially SIADH, first-line measures include fluid restriction, withdrawal of offending medications, and increased solute intake (e.g., oral salt or oral urea) [5,6]. Despite these measures, treatment failure is common. Fluid restriction is often poorly tolerated and ineffective when urine osmolality remains high [4].

Hypertonic saline may achieve rapid correction in acute symptomatic cases, but risks of overcorrection and osmotic demyelination necessitate strict monitoring. In addition, complex or multifactorial etiologies such as coexisting kidney disease, diuretic use, or malignancy-associated ADH secretion frequently contribute to refractory cases that fail standard therapy. To address these challenges, pharmacological agents targeting the vasopressin pathway have been introduced. Vasopressin V₂ receptor antagonists (vaptans) promote aquaresis by blocking ADH activity in the renal collecting ducts. Randomized trials and meta-analyses demonstrate their ability to increase serum sodium more effectively than fluid restriction or placebo, although concerns remain regarding overly rapid correction, cost, and hepatotoxicity [7]. Oral urea has re-emerged as a safe, inexpensive alternative that facilitates osmotic excretion of free water. Recent studies report comparable efficacy to vaptans in SIADH, with fewer serious side effects. Comparative data suggest that while tolvaptan achieves a faster rise in serum sodium, urea remains reliable and well-tolerated in long-term management [8].

Nevertheless, the success of any intervention depends on accurate classification of the hyponatremia subtype. Diagnostic pitfalls, particularly misjudging volume status, frequently contribute to poor outcomes [9]. Guideline comparisons underscore variability in recommended approaches, correction thresholds, and second-line therapy use, further complicating management [10]. Thus, when initial therapy fails, clinicians must reassess the diagnostic framework and escalate treatment in a structured, evidence-based manner. This review aims to decode the management of refractory hyponatremia by synthesizing available evidence on diagnostic pathways and therapeutic strategies. We highlight conventional and emerging treatment modalities, discuss their relative efficacy and limitations, and propose a practical approach to guide escalation when sodium correction fails.

## Review

Materials and methods

Search Strategy

A systematic search was conducted in accordance with Preferred Reporting Items for Systematic Reviews and Meta-Analyses (PRISMA) 2020 guidelines [11]. The search covered PubMed, Embase, Scopus, and the Cochrane Library from inception to December 2024, using a combination of keywords and Medical Subject Headings (MeSH) terms such as “hyponatremia”, “refractory hyponatremia”, “syndrome of inappropriate antidiuretic hormone secretion (SIADH)”, “vasopressin receptor antagonists”, “oral urea”, and “demeclocycline”, with Boolean operators applied to refine results; only peer-reviewed human studies, randomized controlled trials (RCTs), cohort studies, systematic reviews, and guideline-based publications in English were included, while case reports, conference abstracts, animal studies, and narrative reviews were excluded, and reference lists of relevant articles were hand-searched to capture additional evidence.

Eligibility Criteria

Studies were included according to the PICO (Population, Intervention, Comparison, and Outcome) framework [12]: (i) Population: adult or pediatric patients with hyponatremia, including refractory or persistent cases, (ii) Intervention: diagnostic frameworks or therapeutic interventions such as fluid restriction, hypertonic saline, vasopressin receptor antagonists (vaptans), oral urea, or demeclocycline, (iii) Comparators: placebo, standard fluid restriction, guideline-based management, or alternative pharmacologic therapy, and (iv) Outcomes: serum sodium correction, relapse, safety, renal impact, or mortality.

Exclusion criteria comprised case reports, animal studies, conference abstracts, and editorials, in line with established systematic review standards.

Study Selection

Two reviewers independently screened titles/abstracts. Full texts of eligible studies were retrieved and evaluated. Discrepancies were resolved by consensus; the PRISMA diagram documented the process.

Data Extraction

Data extraction was carried out independently by two reviewers using a standardized form to ensure consistency and minimize error. Extracted variables included study design, sample size, patient demographics, underlying etiology of hyponatremia, details of the intervention, comparator groups, and primary and secondary outcomes such as sodium correction, relapse rates, renal impact, and safety profiles. Additional information on pathophysiological findings and long-term follow-up data, where available, was also collected. Discrepancies between reviewers were resolved by consensus or consultation with a third investigator to maintain methodological rigor.

Risk of Bias Assessment

The methodological quality of included studies was evaluated using validated tools appropriate to the study design. RCTs were assessed with the Cochrane Risk of Bias Tool [13], observational cohorts were evaluated with the Newcastle-Ottawa Scale (NOS) [14], guideline syntheses were reviewed using AMSTAR-2 (A Measurement Tool to Assess Systematic Reviews, version 2) [15], and preclinical studies were appraised with SYRCLE (SYstematic Review Centre for Laboratory animal Experimentation)’s Risk of Bias Tool [16]. Each study was assigned a rating of low, moderate, or high risk of bias, with justifications based on study design, sample size, blinding, randomization, outcome assessment, and potential conflicts of interest. This structured approach ensured that both internal validity and external applicability of findings were critically appraised before inclusion in the qualitative synthesis.

Data Synthesis

Because of heterogeneity in study design, populations, and interventions, a qualitative synthesis was undertaken. Data from RCTs, observational cohorts, guideline comparisons, and mechanistic studies were narratively integrated to assess diagnostic strategies and therapeutic outcomes in refractory hyponatremia. The analysis emphasized serum sodium correction, relapse risk, renal safety, and tolerability across different modalities, including fluid restriction, hypertonic saline, vasopressin receptor antagonists, oral urea, and demeclocycline. Findings were organized around the PICO framework to evaluate diagnostic accuracy, treatment efficacy, and safety across included studies.

Ethical Consideration

As this study is a systematic review of previously published data, ethical approval was not applicable. No PROSPERO registration was obtained for this review. All included studies were sourced from peer-reviewed journals, and findings were synthesized in accordance with established methodological standards.

Results

Study Selection Process

**Figure 1:**
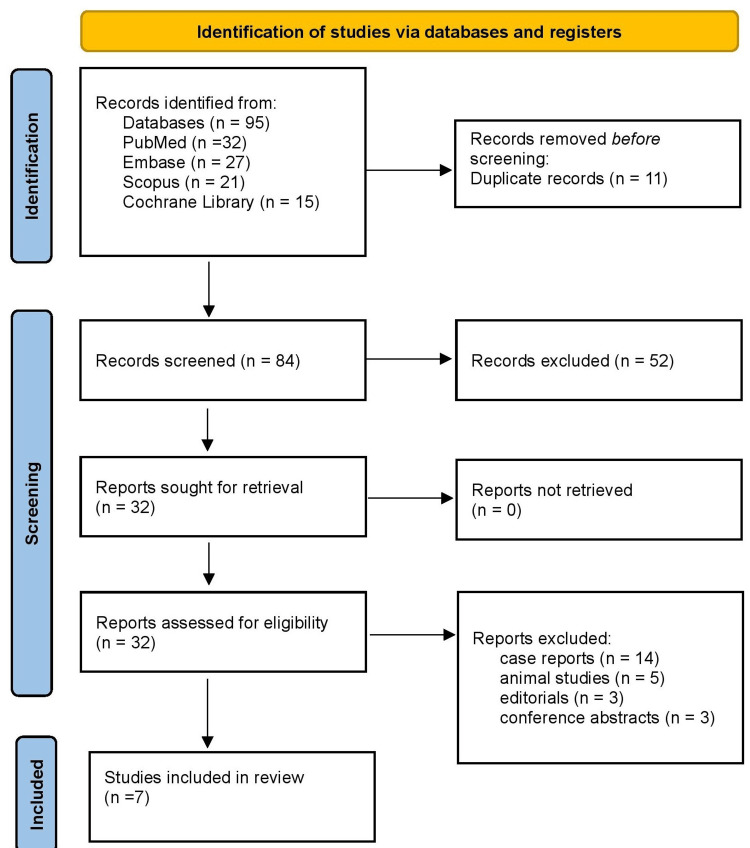
PRISMA 2020 flow diagram PRISMA: Preferred Reporting Items for Systematic Reviews and Meta-Analyses

Figure 1 illustrates the PRISMA flow chart, showing the study selection process. A total of 95 records were identified across four major databases: PubMed (n = 32), Embase (n = 27), Scopus (n = 21), and the Cochrane Library (n = 15). After removal of duplicates (n = 11), 84 records were screened by title and abstract, of which 52 were excluded for irrelevance. A total of 32 full-text reports were sought for retrieval, and all were successfully obtained. Following a detailed assessment, 25 articles were excluded, consisting of case reports (n = 14), animal studies (n = 5), editorials (n = 3), and conference abstracts (n = 3). Ultimately, seven studies met eligibility criteria and were included in the final qualitative synthesis.

Characteristics of the Selected Studies

Table 1 summarizes major clinical and experimental studies on refractory hyponatremia. Tolvaptan showed sustained sodium correction in SIADH through AVP antagonism [17]. Guideline-directed care improved outcomes in mixed hyponatremia but was limited by diagnostic delays [5]. Vaptans were more effective than fluid restriction in oncology patients, though relapses occurred [18]. In heart failure, vaptans provided safe and sustained correction compared with diuretics [19]. Structured diagnostic pathways enhanced accuracy and treatment outcomes in hospitalized adults [20]. Oral urea offered a gradual yet reliable correction in chronic SIADH [6]. Demeclocycline promoted water diuresis by downregulating adenylate cyclase and AQP2 expression in experimental SIADH models [21].

**Table 1:** Characteristics of the Selected Studies SIADH: syndrome of inappropriate antidiuretic hormone; AVP: arginine vasopressin; AQP2: aquaporin-2; mpkCCD: mouse principal kidney cortical collecting duct cells

Authors & Year	Population (P)	Exposure / Condition (I)	Comparator (C)	Outcomes (O)	Pathophysiological Findings	Renal Outcomes	Correction Impact
Verbalis et al., 2010 [17]	110 Adults with SIADH	Tolvaptan therapy	Placebo	Sodium correction, safety	AVP-mediated water retention	Improved water clearance	Significant sustained correction
Spasovski et al., 2014 [5]	Mixed hyponatremia	Guideline-directed management	Standard care	Mortality, recovery	Diagnostic delays frequent	Persistent hyponatremia	Partial correction; frequent relapse
Gralla et al., 2019 [18]	28 Oncology patients with SIADH	Vaptans	Fluid restriction	Response, relapse	Tumor-driven ADH	Preserved renal function	Effective initial correction, relapse in some
Schrier et al., 2006 [19]	448 Heart failure patients	Vaptans	Diuretics	Symptom relief, Na correction	Dilutional pathophysiology	Stable renal function	Sustained correction, safe if monitored
Hoorn and Zietse, 2017 [20]	Hospitalized adults	Diagnostic guideline comparison	Regional/clinical practice	Diagnostic accuracy, treatment outcome	Guidelines harmonize diagnostic pitfalls	Not applicable	Improved correction with structured guideline-based pathways
Soupart et al., 2012 [6]	13 Chronic SIADH patients	Oral urea therapy	Fluid restriction	Sodium rise, tolerability	Osmotic effect via urea excretion	Preserved function	Gradual but reliable correction
Kortenoeven et al., 2013 [21]	In vitro mpkCCD cells + SIADH rodent model	Demeclocycline treatment	Vehicle / untreated	Urine volume, serum sodium, AQP2 abundance	Demeclocycline reduces adenylate cyclase 5/6 expression → lower cAMP → decreased AQP2 transcription	Reduced AQP2 abundance in inner medulla, increased urine output, reduced urine osmolality	Attenuates hyponatremia: increases serum sodium in animal model; supports a water diuresis effect

Risk of Bias Assessment

Table 2 outlines the risk of bias assessment across included studies. The RCTs by Verbalis et al. [17] and Schrier et al. [19] were rated low to low-moderate risk due to robust multicenter randomization and blinding, despite potential funding bias. Spasovski et al.'s study carried a moderate risk given its observational design and possible selection bias, though guideline application strengthened validity [5]. Gralla et al.'s study showed low-moderate risk as it represented a post hoc subgroup analysis, albeit supported by strong parent RCTs [18]. Hoorn and Zietse provided a low-risk synthesis through a comprehensive and transparent guideline-based methodology [20]. The study by Soupart et al. was rated low-moderate risk due to its small sample size but benefited from prospective follow-up [6]. Finally, the study by Kortenoeven et al. carried a moderate risk, reflecting the inherent translational limitations of preclinical animal and cell models despite providing mechanistic clarity [21].

**Table 2:** Risk of Bias Assessment RCT: randomized controlled trial; NOS: Newcastle-Ottawa Scale; AMSTAR-2: A Measurement Tool to Assess Systematic Reviews 2; SYRCLE: Systematic Review Centre for Laboratory Animal Experimentation

Study	Study Design	Risk of Bias Tool	Risk of Bias Rating	Justification
Verbalis et al., 2010 [17]	Randomized Controlled Trial (RCT)	Cochrane Risk of Bias	Low	Placebo-controlled, multicenter design, low attrition
Spasovski et al., 2014 [5]	Guideline-based cohort/observational	NOS	Moderate	Observational, selection bias possible; applied guideline framework
Gralla et al., 2019 [18]	Post hoc subgroup analysis of RCTs (SALT-1/2)	Cochrane	Low-Moderate	Small oncology subgroup, secondary analysis but robust parent RCTs
Schrier et al., 2006 [19]	RCT	Cochrane	Low-Moderate	Industry-funded but rigorous randomization and blinding
Hoorn and Zietse, 2017 [20]	Systematic Review / Guideline Compilation	AMSTAR-2	Low	Comprehensive synthesis across multiple guidelines, transparent methodology
Soupart et al., 2012 [6]	Prospective cohort (small sample)	NOS	Low-Moderate	Small size (n=13), prospective design with consistent follow-up
Kortenoeven et al., 2013 [21]	Preclinical mechanistic study (cell culture + rodent SIADH model)	SYRCLE’s Risk of Bias Tool (animal studies)	Moderate	Preclinical setting, translational limitations, robust mechanistic data

Discussion

Hyponatremia remains the most common electrolyte disorder in clinical practice and continues to challenge physicians when correction fails despite standard approaches. Conventional management is guided by volume assessment. Hypovolemic hyponatremia usually responds to isotonic saline infusion, while hypervolemic states such as heart failure or cirrhosis are managed with fluid and sodium restriction, often combined with loop diuretics. In SIADH and other euvolemic states, fluid restriction and solute supplementation have long served as first-line therapies [22].

However, conventional strategies are often limited by poor patient tolerance (e.g., difficulty adhering to strict fluid restriction), slow onset of effect, and high failure rates-particularly when urine osmolality remains high or the inciting stimulus for ADH secretion persists-resulting in a substantial proportion of patients with refractory hyponatremia who require stepwise, evidence-based escalation of therapy [5,9,20]. Second-line treatments targeting the vasopressin pathway have reshaped the therapeutic landscape. Verbalis et al. demonstrated in a randomized controlled trial that tolvaptan, a selective V₂ receptor antagonist, provides a significant and sustained increase in serum sodium in SIADH patients, surpassing placebo in both efficacy and safety outcomes [17]. Similarly, Schrier et al. confirmed the role of vaptans in hypervolemic hyponatremia related to heart failure, showing symptomatic improvement and stable renal function with careful monitoring [19]. Gralla et al. extended these findings to oncology populations, where vaptans offered superior correction compared with fluid restriction, although relapse remained a concern [18]. Collectively, these studies establish vaptans as a more predictable and effective option than conventional fluid restriction, though cost, availability, and the potential for overly rapid correction remain barriers to universal adoption.

Oral urea has re-emerged as an effective and inexpensive alternative for chronic SIADH. Soupart et al. reported that urea produces gradual but reliable sodium correction with preserved renal function and good long-term tolerability [6]. Compared with tolvaptan, urea induces a slower but steadier rise in sodium, making it particularly useful for long-term outpatient management. In settings where access to vaptans is limited, urea provides an attractive, evidence-based therapy supported by prospective cohort data. Another pharmacological option, demeclocycline, was historically employed for chronic SIADH but has been relegated to a third-line role. Preclinical work by Kortenoeven et al. demonstrated that demeclocycline reduces AQP2 expression via adenylate cyclase inhibition, leading to water diuresis and improved sodium in SIADH models [21]. While mechanistically sound, clinical use is limited by nephrotoxicity, delayed onset, and variable efficacy. These drawbacks restrict its role to selected refractory cases where safer alternatives are unavailable.

Beyond treatment escalation, diagnostic accuracy is pivotal. Misclassification of volume status remains one of the most frequent pitfalls. Spasovski et al. highlighted that guideline-directed management improves outcomes compared with routine care, but also revealed that diagnostic delays and inconsistencies contribute to persistent hyponatremia and relapse [5]. Hoorn and Zietse, in their systematic comparison of international guidelines, emphasized how variability in correction thresholds and second-line recommendations complicates management and creates heterogeneity in clinical practice [20]. Together, these findings stress that successful correction depends not only on therapeutic choice but also on accurate and timely diagnostic frameworks. Several limitations across the evidence base warrant attention. Many of the vaptan trials, while rigorous, were industry-sponsored, introducing potential bias. Oncology subgroup analyses, as reported by Gralla et al., were limited by small sample size and secondary endpoints, reducing external generalizability [18]. Cohort studies such as Soupart et al. [6] were prospective but underpowered, while preclinical data from Kortenoeven et al. cannot be directly extrapolated to clinical care [21]. Guideline comparisons by Hoorn and Zietse reflect methodological transparency but also highlight the absence of universally accepted correction strategies [20]. Overall, while the collective evidence supports escalation beyond fluid restriction, heterogeneity in populations, study design, and outcome reporting continues to limit the strength of pooled conclusions.

Future directions should focus on harmonizing guideline recommendations to reduce variability in practice, particularly regarding thresholds for initiating pharmacological therapy and monitoring strategies for rapid correction. Comparative head-to-head studies of tolvaptan versus urea are urgently needed to establish clear therapeutic hierarchies across different patient populations. Longitudinal follow-up should address relapse, renal outcomes, and patient-centered metrics such as cognitive recovery and functional improvement, which remain underreported in current literature. Moreover, the integration of structured diagnostic algorithms and decision-support systems, as emphasized by Hoorn and Zietse [20], could minimize misclassification and optimize individualized treatment pathways.

## Conclusions

Hyponatremia that fails to correct requires both diagnostic reassessment and escalation of therapy. Conventional measures such as fluid restriction and solute intake are often inadequate, especially in SIADH. Evidence supports the use of vasopressin receptor antagonists, which achieve faster and more predictable correction but carry risks of rapid overcorrection and high cost. Oral urea has re-emerged as an effective, inexpensive, and well-tolerated option, particularly for long-term management. Demeclocycline remains a last-line therapy due to delayed onset and nephrotoxicity. Diagnostic accuracy is equally important, as misclassification of volume status and variability among guidelines continue to hinder outcomes. Future progress will depend on standardized correction thresholds, comparative head-to-head studies of tolvaptan versus urea, and greater focus on patient-centered outcomes such as cognitive recovery and quality of life.
